# The long-term efficacy of nusinersen on respiratory functions in children with symptomatic spinal muscular atrophy type 1

**DOI:** 10.55730/1300-0144.6018

**Published:** 2025-02-02

**Authors:** Mehmet Akif KILIÇ, Fulya KÜREKÇİ, Osman KİPOĞLU, Orhan COŞKUN, Burçin Nazlı KARACEBEY, Selahattin KATAR, Rıdvan AVCI, Hülya Maraş GENÇ, Erkan ÇAKIR, Edibe Pembegül YILDIZ

**Affiliations:** 1 Department of Pediatric Neurology, Faculty of Medicine, İstanbul University, İstanbul, Turkiye; 2 Department of Pediatric Pulmonology, Faculty of Medicine, İstinye University, İstanbul, Turkiye

**Keywords:** Type 1 spinal muscular atrophy, respiratory outcomes, nusinersen efficacy

## Abstract

**Background/aim:**

Available data regarding the effects of nusinersen on respiratory function in the real-world setting are uncertain. We aimed to evaluate the impact of nusinersen on respiratory outcomes over a follow-up period of up to 72 months in patients with symptomatic spinal muscular atrophy type 1 (SMA type 1).

**Materials and methods:**

Respiratory status was defined similarly to previous studies: spontaneous breathing, noninvasive ventilatory support for ≤16 h per day, and permanent assisted ventilation. The planned evaluation time was day 180 (after the fourth dose), day 300 (after the fifth dose), and after the last injection for patients who received nusinersen for 2 years or more.

**Results:**

Our cohort consisted of 32 patients. The mean age at treatment initiation was 6.6 months (range: 2.5–16 months). Twenty-eight of 32 patients were eligible for evaluation after the fourth dose. Twenty-three of 28 patients were eligible for assessment after the fifth dose of nusinersen. Eight *patients* received nusinersen for 2 years or more (range: 26–72 months). At the last assessment, four patients did not require ventilatory support between the ages of 30 and 81 months. One was successfully weaned from invasive ventilatory support after the tenth dose of nusinersen. The respiratory status of most patients remained stable or worsened following the fourth and fifth doses of nusinersen. There were no significant differences in respiratory status between patients who received nusinersen at 6 months of age or younger and those older than 6 months, after the fourth and fifth doses of nusinersen (p > 0.05).

**Conclusion:**

Improvement in respiratory function in patients treated with nusinersen for 2 years or more is generally not expected in the natural course of the disease. However, in our cohort, patients treated with nusinersen mainly maintained their current respiratory status, and many patients required ventilatory support despite treatment with nusinersen. Therefore, our findings may reflect the limited efficacy of nusinersen in symptomatic patients.

## 1. Introduction

Proximal spinal muscular atrophy (SMA) is characterized by progressive muscle weakness and atrophy; it occurs in approximately one in 11,000 live births and is inherited in an autosomal recessive manner [1]. SMA can be categorized into five clinical subtypes (0 to 4) based on the age of symptom onset and the maximum motor function attained [2]. SMA type 1, the most common subtype, refers to infants with symptom onset at 6 months of age or younger who cannot sit unsupported [3,4]. In addition, SMA type 1 is subdivided into three categories based on symptom severity and age of onset: 1A (symptoms in the first 2 weeks of life), 1B (within the third month), and 1C (between 3 and 6 months) [5].

Respiratory complications, which significantly impact morbidity and mortality, are a common concern in SMA patients [2]. The degree of respiratory system involvement correlates with muscle function loss and the type of SMA. Notably, in infants with SMA type 1, the median lifespan without respiratory support has been shown to be less than 2 years [3,4,6]. In SMA patients, diaphragm strength is relatively preserved, while the intercostal muscles exhibit significant weakness [7]. This unequal involvement of the respiratory muscles leads to abnormal asynchronous movement of the ribcage and abdomen. Respiratory muscle weakness manifests as a poor cough reflex and hypoventilation, which in turn lead to inadequate airway clearance, mucus plugging, pneumonia, and ultimately respiratory failure [7–10].

SMA is caused by a homozygous deletion or compound heterozygous mutation in the survival motor neuron 1 (*SMN1*) gene [10]. *SMN1* is responsible for the expression of survival motor neuron (SMN) proteins, which are crucial for the function of motor neurons in the spinal cord and brain stem [10,11]. SMN protein is also encoded by the survival motor neuron 2 (*SMN2*) gene. However, 90% to 95% of the SMN protein encoded by *SMN2* is nonfunctional [12,13]. Most patients with SMA type 1 have two copies of *SMN2* and severe disease phenotype [14]. There is a variation in the number of copies of *SMN2* in individuals without SMA, and unaffected individuals may have no *SMN2* copies [15,16]. However, in patients with SMA, a higher *SMN2* copy number is correlated with a higher level of SMN protein and usually a better phenotype [16,17]. Therefore, modulation of *SMN2* to increase the production of functional SMN protein represents a promising therapeutic approach for SMA [18,19]. Nusinersen is an antisense oligonucleotide that alters the natural course of the disease by modulating *SMN2* [18,19].

Nusinersen is the first disease-modifying drug approved by the US Food and Drug Administration in late December 2016 and by the European Medicines Agency in June 2017 for treating patients with SMA [20,21]. In Türkiye, nusinersen has been reimbursed by the National Health Insurance Fund for patients with SMA since late 2017. Respiratory problems have a significant impact on the prognosis of SMA type 1 and are a common cause of hospitalization, morbidity, and death [7,22]. The relatively short-term effectiveness of nusinersen in respiratory functions has been reported in the literature [23–25]. In this article, we aim to assess the effect of nusinersen on respiratory functions with a follow-up period of up to 72 months in patients with symptomatic SMA type 1.

## 2. Materials and methods

For this single-center, retrospective study, we collected data on symptomatic patients with SMA type 1 who were followed up in Istanbul University’s Department of Pediatric Neurology between November 2017 and January 2024. Patients who had a homozygous deletion or compound heterozygous mutation in *SMN1*, symptom onset at 6 months of age or younger, and inability to sit independently were diagnosed as SMA type 1.

Nusinersen was administered via intrathecal injection. The dosing schedule was planned according to the manufacturer’s recommendations. Nusinersen was intended to be administered on days 1, 15, 30, and 60, followed by maintenance doses at 4-month intervals. Patients were divided into two groups based on age at treatment initiation (Group 1: 6 months of age or younger, Group 2: older than 6 months of age).

Respiratory status is defined similarly to previous studies: spontaneous breathing, noninvasive ventilatory (NIV) support for ≤16 h per day, and permanent assisted ventilation [19,26]. Permanent assisted ventilation is used for patients who need tracheostomy or ventilatory support for ≥16 h per day for >21 continuous days without an acute reversible event [19]. Baseline respiratory status was assessed on the day when the first dose of nusinersen was administered. The planned evaluation times were day 180 (after the fourth dose, the day of the fifth injection), day 300 (after the fifth dose, the day of the sixth injection), and after the last injection for patients who received nusinersen for 2 years or more.

Statistical data were analyzed with SPSS software, version 25.0. Descriptive statistics were used to characterize patient demographics and clinical variables. Quantitative features were reported as mean, standard deviation (SD), median, minimum, and maximum. Categorical variables were reported as frequency and percentage. Quantitative data were compared for paired groups using the Student t-test, chi-square test, and Fisher’s exact test. A p-value of <0.05 was considered significant for all analyses. Informed consent was obtained from patients and/or their parents for the academic study. This retrospective study was approved by the Human Research and Ethics Committees of the Istanbul Medical Faculty (23.11.2022-1397206).

## 3. Results

There were 32 patients in our cohort, 47% (n = 15) of whom were girls. Thirty-one patients had two copies of *SMN2*, whereas one had three copies. There were 22 patients with SMA type 1B, four with type 1A, and six with type 1C. The mean age at treatment initiation (n = 32) was 6.6 months (SD 3.6, range: 2.5–16). Nusinersen was administered to 50% of the patients (n = 16) at 6 months of age or younger. Six of 32 patients (18.7%) died in the study period. Four patients died after the fourth dose, and one patient each died after the 6th and 7th doses. Twenty-eight of 32 patients were eligible for evaluation after the fourth dose. Twenty-three of 28 patients were eligible for assessment after the fifth dose of nusinersen. A total of 226 doses of nusinersen were administered during the study period.

The dosing schedule was planned as mentioned in the method section. However, the planned treatment schedule was delayed due to difficulties in accessing hospitals because of the COVID-19 pandemic, delays in drug supply, and severe infections that prevented the procedure. Additionally, some parents, feeling that the treatment did not meet their expectations, chose to pause it, resulting in further delays in the schedule. Therefore, while the planned completion time for the four loading doses was 60 days, the actual mean completion time was 107.1 days (SD 43.7, range: 63–281) in our cohort. Table 1 presents the respiratory status of the children at baseline and after the fourth dose of nusinersen.

After the fourth dose of nusinersen, 14 patients each in Group 1 and Group 2 were eligible for evaluation (Table 1). There were significant differences between the two groups in terms of the mean age at treatment initiation (3.7 versus 10.1 months) (p < 0.001). Of patients in Group 1, one (7.1%) had an improvement in respiratory status, nine (64.3%) remained stable, and four (28.6%) required further ventilatory assistance after the fourth dose of nusinersen. Of patients in Group 2, none improved, 11 (78.6%) remained stable, and three (21.4%) required further ventilatory assistance after the fourth dose. There were no significant differences in respiratory status between patients in Group 1 and Group 2 after the fourth dose of nusinersen (p > 0.05).

After the fifth dose of nusinersen, 12 patients in Group 1 and 11 in Group 2 were eligible for evaluation (Table 2). There were significant differences between the two groups in terms of the mean age at treatment initiation (3.8 versus 9.8 months) (p < 0.001). Of patients in Group 1, two (14.3%) had an improvement in respiratory status, six (50%) remained stable, and four (33.3%) required further ventilatory assistance after the fifth dose. Of patients in Group 2, none improved, nine (78.6%) remained stable, and two (21.4%) required further ventilatory assistance after the fifth dose. There were no significant differences in respiratory status between patients in Group 1 and Group 2 after the fifth dose of nusinersen (p > 0.05). Eight patients were reviewed in detail for long-term outcomes (2 years or longer). The characteristics of these patients are presented in Table 3.

## 4. Discussion

Nusinersen treatment in patients with SMA type 1 has the potential to improve or stabilize motor function [18,19,27]. However, data regarding respiratory functions are less encouraging, as the need for ventilatory support continues to increase in symptomatic patients with SMA type 1, despite treatment with nusinersen [25,26,28,29]. The available data on the effects of nusinersen on respiratory outcomes in real-world settings remain inconclusive [24,27,30]. Finkel et al. [19] were the first to demonstrate the effectiveness of nusinersen compared to sham control in patients with SMA type 1. Despite significant improvements in motor functions, the improvement in respiratory status in the treated group was not statistically significant. Pane et al. reported no significant change in the respiratory status of patients with SMA type 1 who were treated with nusinersen between baseline and 24 months of follow up [31]. According to Sansone et al., nusinersen may improve respiratory function, especially before 2 years of age [25]. Pechmann et al. reported no significant improvement in respiratory function after 6 months of the nusinersen therapy in symptomatic patients [27]. Compared to the start of treatment, more patients in their study received NIV support or permanent assisted ventilation after 180 days [27]. The age range of patients in their research was broader than in ours. Therefore, we cannot make a head-to-head comparison. Still, in our study, the need for any ventilatory support increased from 46.4% at baseline to 64.3% after the fourth dose of nusinersen (n = 28, planned evaluation time:180 days; mean evaluation time: 223.1 days; Table 1).

In previous studies, the effectiveness of nusinersen on respiratory functions was associated with the age at treatment initiation [24,25]. Our study showed no significant difference in respiratory status between patients who received nusinersen at 6 months of age or younger and those older than 6 months, following the fourth and fifth doses (p > 0.05). There is evidence that initiating treatment mainly during the presymptomatic stage of SMA (before 6 weeks of age) is more effective for respiratory function [32]. In addition, Finkel et al. reported that patients who received nusinersen before 13.1 weeks of age had a higher likelihood of event-free survival (no death or need for permanent assisted ventilation) [19]. Furthermore, LoMauro et al. suggested that the effectiveness of nusinersen on respiratory function depends on the SMA type 1 subtype [33]. Since our cohort consisted of symptomatic patients and only two began treatment before 13.1 weeks of age, we may not have demonstrated a significantly reduced need for ventilatory support among patients who received nusinersen before 6 months of age. Additionally, because most patients in our study were SMA type 1B (n = 22), we could not statistically analyze a possible correlation between disease subtypes.

Ergenekon et al. reported that no improvement in respiratory function was observed on day 180 in any of the patients who received nusinersen [24]. In their study, it was found that among 16 patients who received nusinersen at 6 months of age or younger, five (31.2%) showed worsened respiratory status and 11 (68.8%) maintained their baseline respiratory status on day 180 [24]. In our study, among 14 patients who received nusinersen at 6 months of age or younger, one (7.1%) showed improvement, four (28.6%) had worsened respiratory status, and nine (64.3%) maintained their baseline respiratory status after the fourth dose of nusinersen (planned evaluation time: 180 days, mean evaluation time: 210.9 days, Table 1). Our results for this age group were similar to those of Ergenekon et al. [24]. Another study evaluated the effect of nusinersen in SMA type 1 patients older than 7 months of age and revealed that the need for respiratory support increased significantly over time [34]. According to this study, of 15 patients with two copies of *SMN2*, four (26.7%) had worsened respiratory status, and 11 (73.3%) maintained their baseline respiratory status on day 180 [34]. In our study, among 14 patients who received nusinersen older than 6 months of age, three (21.4%) had worsened respiratory status and eleven (78.6%) remained stable after the fourth dose. Our findings were similar; however, a direct comparison cannot be made due to the broader age range at nusinersen initiation and the exclusion of patients with tracheostomy in that study. Nonetheless, based on these results, we suggest that symptomatic patients with SMA type 1 generally remained stable after the fourth dose of nusinersen, although some continued to experience deterioration in respiratory status.

A study by Sansone et al. found that initiating nusinersen in patients under the age of 7 months did not improve respiratory status on day 300 [25]. According to this research, of 10 patients still alive at day 300 who received their first nusinersen dose under the age of 7 months, respiratory status worsened in seven (70%), while three (30%) remained stable [25]. In our research, of 12 patients who received nusinersen at or under the age of 6 months, four (33.3%) had worsened respiratory status, two (16.7%) had reduced need for ventilatory support, and six (50%) remained stable after the fifth dose (planned evaluation time: 300 days; mean evaluation time: 336.4 days; Table 2). Sansone et al. reported that none of the patients with SMA type 1A showed improvement; however, they did not specify the subtypes of SMA type 1 for patients under the age of 7 months in their study [25]. The differences in worsened respiratory status rates between our study and that of Sansone et al. may be attributed to the SMA type 1 subtype [33].

There were 12 patients in our cohort who were treated with nusinersen for 2 years or longer. Four patients received gene therapy (onasemnogene abeparvovec) before completing 2 years of nusinersen treatment. These patients continued to receive nusinersen for 2 years or longer after gene therapy. The respiratory outcomes of these patients after receiving gene therapy were not included in this study. A more detailed analysis of these patients will be reported separately. Eight patients were evaluated for long-term outcomes (defined as 2 years or longer). Four patients did not require ventilatory support at the time of the last assessment, at ages between 30 and 81 months. Tscherter et al. reported that three of six patients treated before 18 months old did not need ventilatory support at age 15–44 months [35]. In our cohort, one patient (P3) was successfully weaned from invasive ventilatory support following the tenth nusinersen dose. To our knowledge, only two patients have been reported in the literature as having been successfully weaned from invasive ventilatory support following nusinersen therapy [36,37].

Lavie et al. reported the respiratory outcomes of 20 patients with SMA type 1 before and after 2 years of nusinersen treatment [28]. Four patients who did not require ventilation at baseline progressed to assisted ventilation 2 years after nusinersen treatment. All 17 patients who were alive after 2 years of nusinersen treatment required ventilatory support. They reported no improvement in respiratory function in any of the children [28]. In their study, the median age at nusinersen initiation was 13.5 months (range: 1–184) [28]. In our study, four out of eight patients maintained their baseline respiratory status, two required further ventilatory support, and two showed improved respiratory function at the last assessment. The median age at nusinersen initiation for these eight patients in our cohort was 4.6 months (range: 2.5–11). The differences in respiratory outcomes between these studies may be attributed to more advanced disease progression at the time of treatment initiation in the study by Lavie et al. [28].

Our study has several limitations in the interpretation of respiratory outcomes. Although respiratory outcomes were reported in terms of ventilation modality and hours of support in most studies, such assessments may be insufficient to comprehensively evaluate respiratory functions. Additionally, the retrospective design, small cohort size, variations in standards of care, and delays in the treatment schedule—as noted in the results section—may have influenced our findings.

In conclusion, one patient was successfully weaned from invasive ventilatory support, and four did not need ventilatory support at age 30–81 months at the last assessment. It is important to note that improvements in respiratory function after 2 or more years of nusinersen therapy are generally not expected in the natural course of the disease [4,7,38,39]. We observed no significant difference in respiratory status between patients who received nusinersen at 6 months of age or younger and those older than 6 months after the fourth and fifth doses. This lack of difference may be attributed to the fact that both groups were symptomatic. We also found that most patients maintained their respiratory status and many continued to require ventilatory support despite nusinersen therapy. Therefore, our findings may reflect the limited efficacy of nusinersen in symptomatic patients.

## Figures and Tables

**Table 1:** Respiratory status of patients at baseline and after the fourth dose of nusinersen.

	Baseline			After the 4th dose			Total	
	Group 1 (≤ 6 months) n = 14	Group 2 (>6 months) n = 14	p-value	Group 1 (≤ 6 months) n = 14	Group 2 (>6 months) n = 14	p-value	Baseline n = 28	After the 4th dose n = 28
The mean evaluation time (SD, range)	3.7 months(0.7, 2.5–5)	10.1 months (3.4, 6–16)	<0.001	210.9 days* (36.6, 185–270)	236.3 days* (58.6, 192–401)	0.180	6.6 months (3.7, 2.5–16)	223.1 days* (49.2, 185–401)
Spontaneous breathing, n (%)	8 (57.1%)	7 (50%)	0.705	6 (42.9%)	4 (28.6%)	0.430	15 (53.6%)	10 (35.7%)
NIV for ≤ 16 h per day, n (%)	3 (21.4%)	1 (7.1%)	0.596	1 (7.1%)	2 (14.3%)	1.000	4 (14.3%)	3 (10.7%)
Permanent assisted ventilation, n (%)	3 (21.4%)	6 (42.9%)	0.420	7 (50%)	8 (57.1%)	0.705	9 (32.1%)	15 (53.6%)
Worsened respiratory status, n (%)	n.a.	n.a.	n.a.	4 (28.6%)	3 (21.4%)	1.000	n.a.	7 (25%)

Abbreviations: n.a., not applicable; NIV, noninvasive ventilation; SD, standard deviation.

* Respiratory status after the fourth dose was evaluated on the day of the fifth injection. The planned evaluation time was 180 days.

**Table 2:** Respiratory status of the patients at baseline and after the fifth dose of nusinersen.

	Baseline			After the 5th dose			Total	
	Group 1 (≤ 6 months) n = 12	Group 2 (>6 months) n = 11	p-value	Group 1 (≤ 6 months) n = 12	Group 2 (>6 months) n = 11	p-value	Baseline n = 23	After the 5th dose n = 23
The mean evaluation time (SD, range)	3.8 months (0.7, 2.5–5)	9.8 months (3.1, 6–16)	<0.001	336.4 days* (22.2, 303–390)	341 days* (34.4, 312–424)	0.71	6.7 months (3.8, 2.5–13)	338.5 days* (27.8, 303–424)
Spontaneous breathing, n (%)	7 (58.3%)	6 (54.5%)	1.00	5 (41.7%)	4 (36.4%)	1.00	13 (56.5%	9 (39.2%)
NIV for ≤16 hours per day, n (%)	3 (25%)	1 (9.1%)	0.59	2 (16.6%)	1 (9.1%)	1.00	4 (17.4%)	3 (13%)
Permanente assisted ventilation, n (%)	2 (16.7%)	4 (36.4%)	0.37	5 (41.7%)	6 (54.5%)	0.53	6 (26.1%)	11 (47.8%)
Worsened respiratory status	n.a.	n.a.	n.a.	4 (33.3)	2 (18.2)	0.64	n.a.	6 (26%)

Abbreviations: n.a., not applicable; NIV, noninvasive ventilation; SD, standard deviation.

* Respiratory status after the fifth dose was evaluated on the day of the sixth injection. The planned evaluation time was 300 days.

**Table 3:** Characteristics of children who received nusinersen for 2 years or longer.

	Age at nusinersen initiation	SMA subtype	*SMN2* copy number	Total administered nusinersen doses	Age at evaluation after the last nusinersen dose	Follow-up period	Baseline respiratory status	Change from baseline	RS after total administered nusinersen doses
P 1	3.5m	1B	2	8	3y 4m	36m	SB	There was no RS change during the study period.	SB
P 2	2.5m	1B	2	15	4y 9m	54m	NIV for ≤16 h per day	After the 4th dose, transition from NIV for ≤16 h per day to SB.	SB
P 3	11m	1C	2	17	6y 9m	70m	PAV	After the 8th dose transition from PAV to NIV for ≤16 h per day. After the 10th dose transition from NIV for ≤16 h per day to SB.	SB
P 4	3m	1B	2	18	6y 4m	72m	NIV for ≤ 16 hours per day	There was no RS change during the study period.	NIV for ≤16 h per day
P 5	4m	1A	2	11	5y	56m	PAV	There was no RS change during the study period.	PAV
P 6	4m	1B	3	11	3y 8m	40m	SB	After the 6th dose transition from SB to NIV for ≤16 h per day.	NIV for ≤16 h per day
P 7	4m	1B	2	8	2y 6m	26m	SB	There was no RS change during the study period.	SB
P 8	5m	1B	2	8	2y 6m	25m	SB	After the 4th dose transition from SB to PAV.	PAV

Abbreviations: m, months; NIV, noninvasive ventilation; PAV, permanent assisted ventilation; RS, respiratory status; SB, spontaneous breathing; y, years.
